# Medical Surgery

**Published:** 1896-04

**Authors:** G. E. Benninghoff

**Affiliations:** Bradford, Pa.


### MEDICAL SURGERY.

By G. E. BENNINGHOFF, M. D., Bradford, Pa.

THE merging of medicine and surgery into one grand profession was done for a purpose. When it became known that certain drugs would produce the same results as blood-letting, it only seemed proper that he who could do one should be able to do the other, and time has proven the wisdom of such an idea and the association of the two. For all cases where similar results were desired could not safely be treated the one way alone, but it was necessary to decide which of the two ways was the best. The barber of those early days could bleed, but he knew nothing of producing similar results with drugs. The apothecary of that time knew how to do it by drugging his patient, but the sight of blood made him sick. Eventually it became necessary that one should be learned in both, and so came the physician.

As we come down the centuries many things have been changed from the original, either by adding to or taking from, until at the present time there is an effort to draw distinct lines between surgery and medicine, but the association of the two are just as necessary now as they were in times past. There is today as much of a medical side to surgical cases as there ever was, and the rapid progress of surgery, whereby medical cases of a few months ago are now surgical, affords abundance of proof that medical cases have a surgical side. An attempt will be made in this article to show why medicine and surgery should be associated more closely than ever. While so doing, cases demonstrating the above theory will be given, and at the same time an effort will be made to show that we need more physicians who can do blood-letting and drugging at the same time, or on the same patient, so to speak. The distance to step from one to the other is not very far, for the medical man must needs have all, or nearly all, the qualifications of the surgeon. At the present time he is supposed to be able to make a diagnosis of the disease present, to intelligently watch the progress of the case, so that when the danger signal is up he can call in the surgeon, have the surgeon operate, and finally attend the case through to either a successful or fatal termination.

On the other hand, one cannot be a successful surgeon unless he is, first of all, a thorough physician. There has always existed an impression, which undoubtedly originated not in the profession but with the people, that the surgeon must possess some peculiar

Read before the Cattaraugus County Medical Society, November 12, 1895.

make-up, a sort of mythical something, not possessed except by a very select few. One hears great stories regarding a certain surgeon; how he can cut people into pieces without an effort, until as a natural result the people call him a butcher. There is nothing in surgical experience that requires the coldness of mind and heart to the degree, as when one is compelled to sit by and see a patient die for the want of a little surgery. The man who can sit by and coldly wait for death and his fee has more nerve, as it is called, than has any surgeon.

The advocates of specialties claim for the specialist the superlative quality and skill acquired by his extensive experience in that one particular line. But on the other side it can be said, that while in the manual part of his specialty he becomes an expert, generally his powers of reasoning become narrowed until he obtains, so to speak, a special mental faculty, whereby all things partake alone of his particular specialty. It would seem that the broad experienced mind of the all-around general practitioner is better qualified to at least diagnosticate disease than the specialist, provided he has as thorough knowledge as he can have in the completeness of his profession; and further, there are none of the specialties so clearly allied to medicine, except those which are really medical branches, as is surgery, and instead of diverging, as is thought by some of the profession, they are coming more closely together.

This is being brought about because of certain diseases which are primarily medical but later are surgical, also certain surgical conditions which are later medical. Undoubtedly the diagnosis of surgical conditions requires as much skill as does any other part of surgery, yet the license given to the surgeon of the present day, whereby he is at liberty when in doubt to explore into any of the cavities of the body and thereby make his diagnosis certain, make it even more easy than in medical cases.

Fully three-fifths of my surgical work is sent to me by other physicians, and the accuracy of the diagnosis as made by the attending physician has thoroughly proven to me how capable the average physician is to diagnosticate surgical conditions, possibly not in all their detail and fulness, but sufficiently so to denominate the cases as surgical. From the foregoing I do not wish to be understood that all surgical diseases are easy of diagnosis, for many of them are not, but in comparison with medical cases I think they are the easiest.

In surgery the knife reveals many surprises. So does also the test-tube and the microscope in medical as well as surgical cases. One of the first surgical surprises that I found was so interesting to me that I think it will bear relating, as it serves as a good demonstration of the revelations of the knife. A woman had been calling to see me several times, during a period of six months, for a painful enlargement in the right crural canal. The enlargement was oblong, its base being upward and just beneath Poupart's ligament. At that point it was about three-fourths of an inch in diameter and could be reduced by lying down. During the time it was under my observation there was but little change, except in the increasing periods of pain. These were at times intense, but one time, instead of the patient coming to see me, I was sent for. The part had been painful for two days this time and had become very much swollen. There were nausea and vomiting present with extreme exhaustion and constipation. Having previously diagnosticated femoral hernia and at this time being unable to reduce it, I operated to relieve strangulation. After getting down to the sac and incising it I found, very much adhered to it, something which, after freeing it of all adhesions, did not look familiar to me. By continuing steady traction a portion of intestine came into view, which caused me to feel sure that I had the appendix vermiformis. I removed it and the patient recovered. Even after removal it was so enlarged and changed by inflammation and its results that I was uncertain as to what it was; but three years later the lady developed a subserous uterine fibroid, for which I did an abdominal section, and while doing it had a good opportunity to search for the site of the appendix, finding where it had been removed at the former operation.

It is not alone in anomalies that the surgeon meets with surprises, but they often come where the conditions are not abnormal. Only three weeks ago a lady presented herself with an abdominal tumor, which was freely movable and floated about in the abdominal cavity, reaching as high as three inches above the umbilicus. The growth was hard and combined palpation revealed its attachment to the uterus. I did not hesitate to diagnosticate a uterine subserous fibroid. When she was operated on, the tumor was found to be a dermoid cyst of the left ovary, so completely filled that there was no possibility of fluctuation. The tube had adhered to the uterus and formed a sort of secondary pedicle. The tube was much enlarged and contained a fetus one and one-half inches long.

I wish to relate a case in point, to show how easily the surgeon, who relies alone on his surgical experience, can be misled by physical symptoms. Four months ago I was called to see a woman with an abdominal tumor, which was fully six inches in diameter, freely movable, located in the left iliac and lumbar region, extending forward past the median line. One could say it was semi-hard and felt very much like an enlarged spleen, or some form of spleenic tumor, but the physicians attending the case informed me that they had found pus in the urine in large quantities. So they decided, and I agreed with them, that the tumor was an abscess of the kidney. The usual lumbar incision was made over the kidney, through which the abscess and diseased kidney were reached. The patient made a perfect recovery and, with one kidney, is perfectly well. There can be no doubt but that had one depended upon physical symptoms, the abdomen would have been opened and a very serious, if not dangerous, operation would have been undertaken, whereas the one which was done was very simple and comparatively without danger.

But the part of this subject which, from a practical standpoint, interests us most is surgical cases which are medical, either before the advent of the surgeon or after. It is interesting, just at present, to note what is being said regarding the vast number of persons having had, for various diseased conditions, abdominal sections performed that remain invalids. Indeed, it is this fact alone, one may say, that stands as a powerful argument in favor of Pean's method of complete removal of uterus and adnexa, where formerly only the diseased part was removed. The advocates of Pean's method claim that the ratio of complete cures after section is small because the uterus is allowed to remain, and subsequently continues to produce disease either through the nerves or lymphatics. If the surgeon who performed these operations had been a physician, so to speak, the results might have been vastly different.

A surgical operation is so very often done as a last resort, that the idea prevails that it is the last thing that needs to be done. I believe right here is one of the greatest mistakes in the profession. Surgery, at its best, is only the beginning of any required treatment for the cure of disease, and is only a small part of it. Why a patient, who has been suffering for from one to twenty years from some chronic surgical condition, should be considered cured when the surgeon has removed the sutures, is one of the most incompatible ideas which we are compelled to analyse. Take, for instance, any form of periuterine inflammation, which results in pus formation or extensive adhesions, from which a woman has been suffering for years. Is it not the invariable rule that the nervous system is a wreck? Spinal irritation, with all the host of symptoms that go with that condition, indigestion of all forms, cardiac irritations; in short, there are so many sympathetic disturbances, that the patient hardly thinks of the original symptoms until reminded of them by her physician. The part originally diseased is removed, but all the other semi-diseased nerves and organs are allowed to remain and get well of themselves. Is it strange that often they do not get well?

In my surgical work I have never ceased to remember that I am a physician and I have always thought it proper to treat patients medically before and after operation. Sometimes, if well treated before intended operations, the surgeon's occupation will be gone, because the case will have gotten well. Generally, when the physician attending a surgical case becomes convinced that an operation is necessary, he sends his patient to the surgeon who performs the operation. If the patient recovers from it she is so thankful for her life, that she sings praises of the surgeon. Not that she is cured, but because she did not die in the great ordeal through which she passed. When, after a few months, many of the old symptoms remain, she either goes to see the surgeon, or she goes direct to her own physician. The latter is disgusted when she relates the same old symptoms and quickly prescribes for her and prescribes her out of his office. Thus she passes on, from year to year, uncured, and she is numbered with those who receive no benefit from a surgical operation which was really necessary. Had her physician and surgeon been one and the same, the treatment would have been continued until she was cured. And what holds good in this hypothetical case, is true in very nearly all operative surgical cases.

Consider the legion of tubercular diseases which come under the surgeon's hand, and all allied affections. Do they not require the intelligent treatment of the physician in order to be cured? Think, too, of the rachitic! in these what can surgery do without medicine? Several times rachitic cases have come under my care that have undergone surgical operations for deformities. All treatment ceased at the advent of the surgeon and was not begun again. The result was that the deformity returned worse than ever. In reference to the importance of medical treatment with surgical operations, I wish to offer the results as to cure in 120 abdominal sections, performed prior to 1895. Those made since the beginning of this year are not included, because the time since doing the work is too short to determine whether they are or are not cured. These 120 were done from 1889 to 1895 and for all conditions. There were 13 deaths, leaving 107 cases which recovered from the operation, all of which required medical treatment either before or after the operation. In this list, if two are excluded who have ventral hernias, but are otherwise well, there are only two who are not cured, but even they are greatly benefited and both of these had ureter disease prior to operation. Thus of these 107 cases, 105 were cured, and compared with what is claimed as a result in these operations I am led to believe that medical treatment cured many of them, which without it would have been surgical failures, and it is these and similar results in other surgical conditions which force me to believe that the physician and surgeon should be one.

Is it going too far for us to say that a patient to receive the proper care prior to, and after a surgical operation, should be operated upon by the physician? It will not be a great while until the thorough instructions that the medical student is now obliged to receive will be such to thoroughly equip him to do his own surgery, quite as well as to treat disease medically. Even now our great medical colleges show the student more surgery than medicine. Why, then, should he not do both? That the time is not far distant when he will, is demonstrated very plainly and when he does many lives will be saved by his promptness to operate at the golden moment, instead of waiting for the surgeon.

